# The Concept of Accuracy Analysis of the Vertical Displacements Gained from the Hydrostatic Levelling Systems’ Measurements

**DOI:** 10.3390/s21144842

**Published:** 2021-07-15

**Authors:** Waldemar Kamiński

**Affiliations:** Faculty of Civil and Environmental Engineering, Gdańsk University of Technology, 80-233 Gdańsk, Poland; waldemar.kaminski@pg.edu.pl

**Keywords:** hydrostatic levelling systems, vertical displacements, covariance matrices, mean errors of displacements

## Abstract

Nowadays, hydrostatic levelling is a widely used method for the vertical displacements’ determinations of objects such as bridges, viaducts, wharfs, tunnels, high buildings, historical buildings, special engineering objects (e.g., synchrotron), sports and entertainment halls. The measurements’ sensors implemented in the hydrostatic levelling systems (HLSs) consist of the reference sensor (RS) and sensors located on the controlled points (CPs). The reference sensor is the one that is placed at the point that (in theoretical assumptions) is not a subject to vertical displacements and the displacements of controlled points are determined according to its height. The hydrostatic levelling rule comes from the Bernoulli’s law. While using the Bernoulli’s principle in hydrostatic levelling, the following components have to be taken into account: atmospheric pressure, force of gravity, density of liquid used in sensors places at CPs. The parameters mentioned above are determined with some mean errors that influence on the accuracy assessment of vertical displacements. In the subject’s literature, there are some works describing the individual accuracy analyses of the components mentioned above. In this paper, the author proposes the concept of comprehensive determination of mean error of vertical displacement (of each CPs), calculated from the mean errors’ values of components dedicated for specific HLS. The formulas of covariances’ matrix were derived and they enable to make the accuracy assessment of the calculations’ results. The author also presented the subject of modelling of vertical displacements’ gained values. The dependences, enabling to conduct the statistic tests of received model’s parameters, were implemented. The conducted tests make it possible to verify the correctness of used theoretical models of the examined object treated as the rigid body. The practical analyses were conducted for two simulated variants of sensors’ connections in HLS. Variant no. I is the sensors’ serial connection. Variant no. II relies on the connection of each CPs with the reference sensor. The calculations’ results show that more detailed value estimations of the vertical displacements can be obtained using variant no. II.

## 1. Introduction

The determination of vertical and horizontal displacements of the engineering objects is one of the main tasks of engineering geodesy. While determining the displacements, different measuring technologies can be used, e.g., GNSS systems, terrestrial and airborne laser scanning, interferometric synthetic aperture radar (InSAR), electronic tachymeters, precise levelling, hydrostatic levelling systems. From the methods mentioned above, the special importance for the vertical displacements’ determination is played by the HLS. The HLS rule comes from the Bernoulli’s law that is most often presented in the following form:

(1) $$\frac{1}{2}\rho v^{2}+P+\rho g\bar{h}=const,$$

where: v—liquid flow speed, P—hydrostatic pressure, ρ—liquid density, g—acceleration of gravity, $\bar{h}$—height of liquid column (reading from sensor).

Assuming that for the majority of HLS the liquid is at rest (v = 0), Equation (1) takes the simplified form

(2) $$P+\rho g\bar{h}=const.$$

The HLS’s special importance is that it enables to determine the vertical displacements with the accuracy order of 0.01 mm. Hence, the HLSs are mainly used for highly precise measurements concerning the engineering objects’ monitoring, e.g., tunnels, dams, building objects, production halls. In order to gain very precise and reliable results, the corrections that cause the heights’ changes $\bar{h}$ of the liquid used in HLSs, should be taken into account in the observations’ results. The changes of liquid heights $\bar{h}$ resulting from the Bernoulli’s law can be caused by

1. The change of liquid density ρ caused mainly by the temperature change *T* (e.g., [1,2,3,4,5]): $\vartheta_{1}$;
2. The changes of the atmospheric pressure P between system’s sensors (e.g., [3,6]): $\vartheta_{2}$;
3. The change of the acceleration of gravity (e.g., [1]): $\vartheta_{3}$.The HLSs’ accuracy is determined also by other factors of which values have to be taken into account in heights readings $\bar{h}$ of system’s sensors, inter alia;
4. Tidal phenomenon (low and high tides): $\vartheta_{4}$. While designing HLS, where the overcoming of water fording is planned, the tidal effect has to be taken into account, (e.g., [1,5,7]);
5. The systematic errors: s. The proposal of elimination of systematic errors from HLSs is presented inter alia in paper [8];
6. The influence of dynamic factors: $\vartheta_{5}$. Using HLSs for monitoring of buildings’ foundations, where working machines and other devices can cause vibrations of whole system, e.g., [7];
7. The influence of electrostatic field: $\vartheta_{6}$. The effect of electrostatic field with specified intensity can cause the phenomenon of liquid level change in system’s sensors. This influence will be significant when the difference mentioned above appears in the neighbourhood of particular system’s sensors [9];
8. Correction: $\vartheta_{7}$ which takes into account other possible factors that have influence on the final reading value h, e.g., [3].

On the basis of consideration presented above, the following deterministic model can be formed:

(3) $$h=\bar{h}+\sum_{i=1}^{7}\beta_{i}\vartheta_{i}+s+g+\varepsilon ,$$

where: h—reading from sensor after corrections, β—coefficient (β=1—when correction is included, β=0—without corrections), s—systematic error, g—outliers (gross error), ε—error of sensor’s measurement, $\bar{h}$—direct reading from measured sensor (row data). It has to be mentioned that gross error g and systematic error s can be eliminated while HLSs’ calibration (e.g., [7]). 

Introducing the corrections $\vartheta_{i}$ to the readings $\bar{h}$ (all or chosen, depended on the HLS’s configurations and used sensors) enables to determine the heights difference dZ between sensors i=1,…,n, (n—number of sensors) from the following formula:

(4) $$dZ=Z_{i+1}-Z_{i}=h_{i}-h_{i+1}.$$

**Figure 1:**
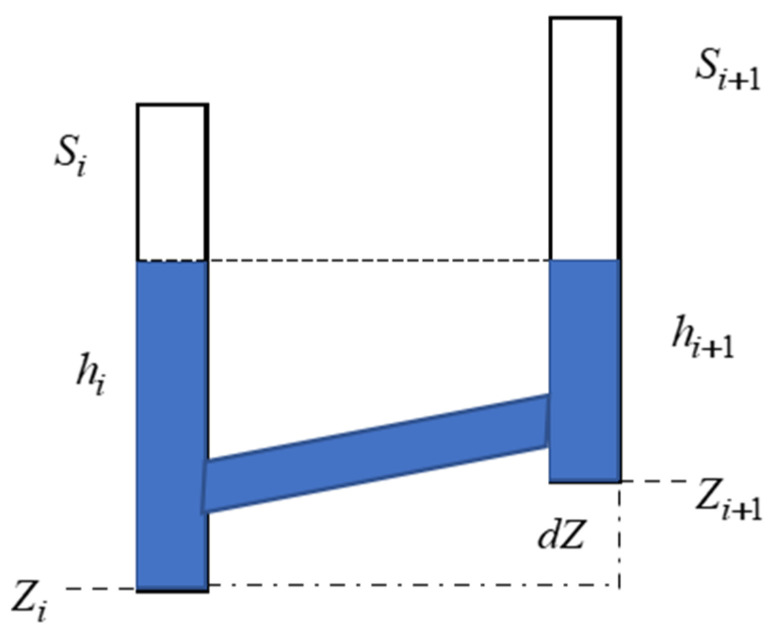
The HLS rule.

The graphical illustration of Equation (4) is schematically presented on Figure 1.

The following notation in Figure 1 was assumed: $S_{i,}\;S_{i+1}$—sensors’ numeration, $h_{i},\;h_{i+1}$—readings of raw data after corrections, $Z_{i},\;Z_{i+1}$—CPs’ heights, dZ—heights’ difference of CPs. 

The influence of corrections implemented to the calculations and mentioned above, should also be included while providing the accuracy assessment of whole HLS. For this purpose, the values of mean errors (respondent to used corrections) have to be also taken into account. In the works mentioned above as well as in others papers of the subject’s literature, only the individual influence of mean errors of the corrections ϑ mentioned above are analysed. There are not any final solutions regarding the comprehensive problem’s approach to accuracy assessment of the vertical displacements’ values gained from all corrections implemented in HLS (at least the author did not find any). In this paper, the comprehensive accuracy analysis is understood as the usage of all mean errors’ values related to corrections ϑ that influence on the HLS’s accuracy. Hence, the reading’s accuracy can be presented in the following form:

(5) $$m_{h}^{2}=m_{\bar{h}}^{2}+m_{P}^{2}+m_{g}^{2}+m_{\rho }^{2}+m_{TP}^{2}+m_{E}^{2}+m_{D}^{2}+m_{R}^{2},$$

where: $m_{h}$—mean error of reading from sensor after corrections $m_{\bar{h}}$—mean error of the reading from HLS’s sensor before corrections, $m_{P}$—mean error of atmospheric pressure, $m_{g}$—mean error of acceleration of gravity, $m_{\rho }$—mean error of liquid density used in HLS, $m_{TP}$—mean error of tidal effect, $m_{E}$—mean error of electrostatic field’s influence, $m_{D}$—mean error of dynamic factors, $m_{R}$—residual mean error taking into account other possible mean errors coming from implementation of corrections $\vartheta_{i}$.

In this paper, the corrections mentioned above as well as the system’s mean errors coming from implemented corrections, will not be analysed. These issues, because of their wide range and a lot existing papers, will be the subject of other author’s works. In this work, the author presents only the concept that enables the comprehensive accuracy assessment using as example Equation (5) or its changed (extended or limited) formula depending on the HLS’s configuration. In Section 2, the theoretical basics of proposed concept are described. The examples of practical implementation are presented in Section 3. The calculations and accuracy analysis were conducted for two variants of HLS’s sensors connection. Variant no. I is the HLS’s sensors’ serial connection. While variant no. II is the connection of each sensor located on CP with the reference sensor. The discussion about the calculations’ results is provided in Section 4. Summing up the results, it must be stated that for all examined variants, the same values of CPs’ vertical displacements were obtained. These two variants, however differ from each other regarding the accuracy assessment of final determination of vertical displacements $d_{i}$, (*i* = 1,…,*n*). While analysing the results, it is noticeable that in variant no. II more precise assessment of vertical displacements than in variant no. I was gained. The attention should also be paid to the form of cofactors’ matrix of vertical displacements $Q_{d}$ in variant no. I, where there are covariance values that enable to determine the correlation of system’s components. At the same time, the form of cofactors’ matrix $Q_{d}$ in variant no. II shows no correlation. On the basis of the calculations made, the following general conclusion can be defined: taking into account the accuracy of determinations $d_{i}$, variant no. II is better solution. The analyses presented in this work do not fully describe the issues considered in this paper (as it was mentioned above) and they will be the subject of author’s future works. 

## 2. Theoretical Foundations

The HLS’s essence (e.g., [5,10]) relies on the determination of heights’ difference between system’s sensors for each measurement epoch. Assuming that $\;h_{i}$—is the reading (after implementing necessary corrections ϑ) from sensor *i* (*i* = *RS*,1,2,…*n*; *n*—number of sensors located on CPs), it can be written as follows:

(6) $$dZ_{k}=h_{i}-h_{i+1}=Z_{i+1}-Z_{i},\;k=1,\ldots ,n.$$

Denoting the vector of heights’ difference as $y={\left[dZ_{1},\ldots ,dZ_{k},\ldots ,dZ_{n}\right]}^{T}$ and parameters’ vector (CPs’ heights) as $X={\left[Z_{1},\ldots ,Z_{n}\right]}^{T}$, the following formula can be written:

(7) AX=y,

and

(8) $$X=A^{-1}y,$$

where: A—the known coefficients’ matrix.

The X parameters are determined for each measurement epoch *j* = 0, 1,…, *m*; *m*—number of measurement epoch. Hence, the CPs’ vertical displacements can be determined as follows:

(9) $$d=X^{j}-X^{j=0},$$

where: $X^{j=0}$—vector of CPs’ locations at starting (original) epoch *j* = 0, to which the results from next measurements epochs will be related, $X^{j}$—vectors of CPs’ locations at epoch *j* = 1,…*m*, and $d={\left[d_{1},\ldots ,d_{n}\right]}^{T}$—vector of CPs’ vertical displacements. 

The law of errors’ propagation, formulated most often in the following form, is used for conducting the accuracy analyses

(10) $$Q=DQ_{L}D^{T},$$

where: D—known transformation matrix, $Q_{L}$—cofactors’ matrix (variances’ approximations) of observations’ results, Q—searched cofactors’ matrix. 

Using Equation (10), the cofactors’ matrix $Q_{X}$ of X vector can be determined from the following relation (assuming that $D=A^{-1}$, and $Q_{L}=Q_{y}$ and $Q_{y}=P_{y}^{-1},\;P_{y}=Q_{y}^{-1},\;P_{y}$—weights’ matrix)

(11) $$Q_{X}=A^{-1}P_{y}^{-1}{\left(A^{-1}\right)}^{T}={\left(A^{T}P_{y}A\right)}^{-1}={\left(A^{T}Q_{y}^{-1}A\right)}^{-1}.$$

Here, the important problem is the determination of cofactors’ matrix $Q_{y}$ that is related to corrections ϑ.

The Bernoulli’s law that is used in the hydrostatic levelling can be also presented in the following form

(12) $${\bar{h}}_{i}+P_{i}/(g_{i}\rho_{i})=dZ_{k}+{\bar{h}}_{i+1}+P_{i+1}/(g_{i+1}\rho_{i+1}),$$

where: ${\bar{h}}_{i},{\bar{h}}_{i+1}$—readings gained from sensors respectively $S_{i},S_{i+1}$, $P_{i},P_{i+1}$—values of atmospheric pressure, $g_{i},g_{i+1}$—values of acceleration of gravity, $\rho_{i},\rho_{i+1}$—liquid density.

Transforming the Equation (12) the result is

(13) $$dZ_{k}={\bar{h}}_{i}+P_{i}/(g_{i}\rho_{i})-{\bar{h}}_{i+1}-P_{i+1}/(g_{i+1}\rho_{i+1}).$$

While determining the values of mean errors $m_{dZ}$ of differences $dZ_{k}$ from Equation (13), the following relation is obtained

(14) $$\begin{array}{l} m_{dZ}^{2}={\left(\frac{\partial dZ}{\partial {\bar{h}}_{i}}\right)}^{2}m_{\bar{h}i}^{2}+{\left(\frac{\partial dZ}{\partial P_{i}}\right)}^{2}m_{Pi}^{2}+{\left(\frac{\partial dZ}{\partial g_{i}}\right)}^{2}m_{gi}^{2}+{\left(\frac{\partial dZ}{\partial \rho_{i}}\right)}^{2}m_{\rho i}^{2}+\\ +{\left(\frac{\partial dZ}{\partial {\bar{h}}_{i+1}}\right)}^{2}m_{\bar{h}i+1}^{2}+{\left(\frac{\partial dZ}{\partial P_{i+1}}\right)}^{2}m_{Pi+1}^{2}+{\left(\frac{\partial dZ}{\partial g_{i+1}}\right)}^{2}m_{gi+1}^{2}+{\left(\frac{\partial dZ}{\partial \rho_{i}+1}\right)}^{2}m_{\rho i+1}^{2} \end{array}$$

where: $m_{\bar{h}\;i},m_{\bar{h}\;i+1}$—values of mean errors of measuring sensors’ readings, $m_{Pi},m_{Pi+1}$—values of mean errors of atmospheric pressure’s readings, $m_{gi},m_{gi+1}$—values of mean errors of acceleration of gravity, $m_{\rho i},m_{\rho i+1}$—values of mean errors of liquid density used in HLS. It is worth to add that the liquid density depends mainly on the temperature value *T* (what was mentioned before), hence the values of mean errors of liquid density $m_{\rho }$, will be related to the mean errors of temperature determination $m_{\rho }≅m_{T}$ ($m_{T}$—mean error of temperature determination *T*). 

The values of mean errors—$m_{\bar{h}}$, $m_{P}$, $m_{g}$, $m_{\rho }$—can be obtained on the basis of the accuracy analyses conducted earlier (while the HLS’s testing) or from the technical specifications of the measuring equipment used. While conducting the accuracy analyses $dZ_{k},\left(k=1,\ldots ,n\right)$ using Equation (13) then Equation (14) can be implemented. Supposing that the HLS’s sensors are placed with the distances that enables to accept the assumption about identical parameters: $P_{i}=P_{i+1},\;g_{i}=g_{i+1},\;\rho_{i}=\rho_{i+1}$, then Equation (13) after implementing essential corrections ϑ can be written in the following form

(15) $$dZ_{k}=h_{i}-h_{i+1}.$$

In this paper, the author does not decide for which distances between HLS’s sensors this simplification can be used. The implementation of simplification mentioned above, should come from the individual theoretical assumptions, dedicated for specific engineering object. 

Accepting the simplification described in Equation (15) and looking for the values of mean errors $m_{dZk}$, the following formula is obtained

(16) $$m_{dZk}^{2}={\left(\frac{\partial dZ}{\partial h_{i}}\right)}^{2}m_{hi}^{2}+{\left(\frac{\partial dZ}{\partial h_{i+1}}\right)}^{2}m_{hi+1}^{2}.$$

The values of mean errors $m_{dZk}$ gained from Equation (14) or Equation (16) enable to create the cofactors’ matrix $Q_{y}$ in the following form

(17) $$Q_{y}=diag(m_{dZ1}^{2},\ldots ,\;m_{dZk}^{2},\ldots ,m_{dZn}^{2}),$$

(diag—diagonal matrix).

Assuming that, in epochs *j* = 0, 1,…*m*, the same measuring sensors are used, the values of cofactors’ matrix is $Q_{y^{j=0}}=Q_{y^{j}}=Q_{y}$. The $X^{j=0}$ values gained from Equation (8) with the cofactors’ matrix $\left(Q_{X^{j=0}}={\left(A^{T}Q_{y}^{-1}A\right)}^{-1}\right)$ and $X^{j}$ with cofactors’ matrix $\left(Q_{X^{j}}={\left(A^{T}Q_{y}^{-1}A\right)}^{-1}\right)$ enable to determine the vector of vertical displacements $d=X^{j}-X^{j=0}$ from Equation (9). For the displacements $d=X^{j}-X^{j=0}$ obtained in such way, the cofactors’ matrix $Q_{d}$ can be determined from the form

(18) $$Q_{d}=Q_{X}{}_{j=0}+Q_{X}{}_{j}.$$

Using Equation (18), the mean errors of the CPs’ vertical displacements can be determined in the following relation

(19) $$m_{di}=\sqrt{{\left(Q_{d}\right)}_{i,i}},\;i=1,\ldots ,n,\;n\text{—}\mathrm{number}\;\mathrm{of}\;\mathrm{CPs}\;$$

${\left(Q_{d}\right)}_{i,i}$—diagonal element of cofactors’ matrix $Q_{d}$.

Using the calculated values of vertical displacements $d={\left[d_{1},\ldots ,d_{n}\right]}^{T}$, the modelling process of the whole examined object (treated as rigid body) can be conducted. The polynomials can be used in the modelling process. The polynomial equation, adapted to the subject of the vertical displacement’s determination, can be written in the following form) [11,12].

(20) $$d_{i}=a_{0}+a_{1}X_{i}+a_{2}Y_{i}+a_{3}X_{i}^{2}+a_{4}Y_{i}^{2}+a_{5}X_{i}Y_{i}+\ldots ,$$

where: $a_{0},\;a_{1},\;a_{2}\ldots$—coefficient of polynomial, $X_{i},\;Y_{i}$—CPs rectangular coordinates, i=1,…,n—number of controlled points.

The subject of selection of the best determination models was described inter alia in work [13] and will not be analyzed deeply in this paper. This process should be done individually for specific examined objects. Thus in further empirical analyses, Equation (21) was used for modelling. This equation is often used in the subject of coordinates transformation. The dependence adapted for the purpose of this work enables to treat the examined objects as the rigid body. Hence

(21) $$d_{i}=T_{Z}+X_{i}\varepsilon_{Y}-Y_{i}\varepsilon_{X},$$

where parametes: $T_{Z}$—vector of translation of the vertical system’s origin along the vertical axis *Z*, $\varepsilon_{Y}$—rotation angle around the Y axis, $\varepsilon_{X}$—rotation angle around X axis. Coefficients $T_{Z},\;\varepsilon_{Y},\;\varepsilon_{X}$—can be determined using the traditional method of the least squares (LS).

Marking parameters’ vector as $t={\left[T_{Z},\;\varepsilon_{Y},\;\varepsilon_{X}\right]}^{T}$ corrections’ vector $\delta ={\left[\delta_{1},\ldots ,\delta_{n}\right]}^{T}$ calculating for displacements $d={\left[d_{1},\ldots ,d_{n}\right]}^{T}$ and the coefficients’ matrix $H=\left[\begin{array}{l} 1,X_{1},Y_{1}\\ \cdots \cdots \cdots \cdots \\ 1,X_{n},Y_{n} \end{array}\right]$ the corrections’ equation can be presented in the following form

(22) δ=Ht+d.

The objective function will take here the following formula

(23) $$\psi =\delta^{T}P_{d}\delta =\min,$$

where: $P_{d}=Q_{d}^{-1}$—matrix of wages determined using the vertical displacements $\left(P_{d}^{-1}=Q_{d}\right)$. The solution to this problem (23) using the LS method is the following vector

(24) $$\hat{t}=-{\left(H^{T}P_{d}H\right)}^{-1}H^{T}P_{d}d.$$

Determining the cofactors’ matrix $Q_{\hat{t}}$ of $\hat{t}$, vector, the following formula is gained

(25) $$Q_{{}_{\hat{t}}}={\left(H^{T}P_{d}H\right)}^{-1}.$$

The corrections’ vector

(26) $$\hat{\delta }=H\hat{t}+d,$$

with the cofactors’ matrix

(27) $$Q_{\hat{\delta }}=P_{d}-H{\left(H^{T}P_{d}H\right)}^{-1}H^{T}.$$

As a result of calculating the covariance’s factor, the following form is obtained

(28) $$m_{0}^{2}=\frac{{\hat{\delta }}^{T}P_{d}\hat{\delta }}{f},$$

where $f=n-m_{r}$—number of redundancy observations, $m_{r}$—number of parameters for adopted model (in this paper $t={\left[T_{Z},\;\varepsilon_{Y},\;\varepsilon_{X}\right]}^{T}$, hence $m_{r}=3$). The variance’s coefficient $m_{0}^{2}$ can be used in order to verify the correctness of adopted model of vertical displacements of examined object. In order to verify the correction of the deformation model and selection of the best model gained from the vertical displacements, the procedures used for statistical hypothesis verification can be adopted. Calculated coefficient $m_{0}^{2}$ is tested here. The global test relies on the two alternative hypothesises verification: zero hypothesis

(29) $$H_{0}:{\hat{\sigma }}_{0}^{2}=m_{0}^{2},$$

and the alternative hypothesise

(30) $$H_{1}:{\hat{\sigma }}_{0}^{2}\neq m_{0}^{2},$$

where ${\hat{\sigma }}_{0}^{2}$—is the estimator of covariance coefficient, common for both measurement epochs. In order to verify two alternative hypothesises the assumption about the vector distribution $\delta ={\left[\delta_{1},\ldots ,\delta_{n}\right]}^{T}$ has to be adopted. Assuming that the displacements’ errors have normal distribution or that they were transformed to normal distribution using for example the Box-Cox transformation, the testing statistic $T_{G}$ is determined from the following form

(31) $$T_{G}=\frac{{\hat{\sigma }}_{0}^{2}}{m_{0}^{2}}∼F_{\alpha }\left(r,\nu \right),$$

where $F_{\alpha }$—is the critical value, read from the *F*-Snedecora distrubution’s tables for adopted significance level-α (typically: α=0.05 or α=0.01), r, ν—number of degrees of freedom. In order to verify the hypothesis $H_{0}:{\hat{\sigma }}_{0}^{2}=m_{0}^{2}$ the global test $T_{G}$ can be conducted, where for ${\hat{\sigma }}_{0}^{2}=s_{0}^{2}$ the statistics [12,14] can be used.

(32) $$\begin{array}{l} T_{G}=\frac{s_{0}^{2}}{m_{0}^{2}}∼F_{\alpha }\left(r,\nu \right)\\ s_{0}^{2}=\frac{f_{\left(j=0\right)}s_{0(j=0)}^{2}+f_{j}s_{0j}^{2}}{f_{\left(j=0\right)}+f_{j}}=\frac{q}{f}\\ q={\left(\upsilon^{T}P\upsilon \right)}_{\left(j=0\right)}+{\left(\upsilon^{T}P\upsilon \right)}_{j};f=f_{\left(j=0\right)}+f_{j} \end{array}$$

where:$\;f_{\left(j=0\right)},\;f_{j},\;f$—degrees of freedom respectively in epochs j=0, j, and degrees of freedom sum (f), υ—corrections’ vector for observations respectively in epoch j=0, as well as j. However, it has to be noted that the measurements’ results were not aligned either in epoch j=0 or in epoch j. Hence, there are no coefficients’ values $s_{0(j=0)}^{2},\;s_{0j}^{2}$. Thus, using the test $T_{G}$ (Equation (32)) meets the difficulties. 

Hence, there is the need for derivation of the coefficient form ${\hat{\sigma }}_{0}^{2}$ adequate to the analyzed problem. The coefficient ${\hat{\sigma }}_{0}^{2}$ will then be used for statistic $T_{G}$ calculations. According to this it can be assumed that CPs are located on the horizontal space of examined object treated as the rigid body. Hence, the theoretical values of vector $t={\left[T_{Z},\;\varepsilon_{Y},\;\varepsilon_{X}\right]}^{T}$ will be equal to $T_{Z}=0,\;\varepsilon_{Y}=0,\;\varepsilon_{X}=0$, respectively. After the points’ stabilization and measurements made in epoch j, the following values were gained ${\hat{T}}_{Z}{}^{j},\;{\hat{\varepsilon }}_{Y}{}^{j},\;{\hat{\varepsilon }}_{X}{}^{j}$ that are different from the theoretical values. Therefore, the following formula can be written ${\hat{t}}^{j}={\left[{\hat{T}}_{Z}{}^{j},{\hat{\varepsilon }}_{Y}^{j},{\hat{\varepsilon }}_{X}^{j}\right]}^{T}$. Assuming that the random error of estimator $\xi =\hat{t}-t$, hence $\hat{t}=t+\xi$. Because $E(\hat{t})=t$, so $E(\xi )=E(\hat{t})-t=0$ and $C_{\xi }=C_{\hat{t}}$. The covariances’ matrix $C_{\hat{t}}=\sigma_{0}^{2}Q_{\hat{t}}$, where $Q_{{}_{\hat{t}}}={\left(H^{T}P_{d}H\right)}^{-1}$ is a cofactors’ matrix defined earlier in Equation (25). There are also the dependences: $Q_{{}_{\hat{t}}}=P_{\hat{t}}^{-1},P_{\hat{t}}=Q_{\hat{t}}^{-1}=H^{T}P_{d}H$. Thus, the result is, that if $\hat{t}∼N\left[t,C_{\hat{t}}\right]$ (N—normal distribution) then the difference $\xi =\hat{t}-t$ also has normal distribution $\xi ∼N\left[E(\xi )=0,C_{\hat{t}}\right]$(E(ξ)—expected value). What appears from the theory of square forms is that the square form $\xi^{T}\Theta \xi$ has the distribution $\chi_{f1,f2}^{2}$ with parameters $f_{1}=\mathrm{Tr}(\Theta C_{\hat{t}})$, $f_{2}={\left[E(\xi )\right]}^{T}\Theta E(\xi )$ (Tr—matrix trace). The matrix Θ has to, however, fulfill one condition: $\Theta C_{\hat{t}}\Theta =\Theta$. Such matrix is matrix $\Theta =H^{T}C_{d}^{-1}H$, because $\Theta C_{\hat{t}}\Theta =H^{T}C_{d}^{-1}H{\left(H^{T}C_{d}^{-1}H\right)}^{-1}H^{T}C_{d}^{-1}H=\Theta$. Determining

(33) $$f_{1}=\mathrm{Tr}(\Theta C_{\hat{t}})=\mathrm{Tr}\left[H^{T}C_{d}^{-1}H^{T}{\left(H^{T}C_{d}^{-1}H\right)}^{-1}\right]=\mathrm{Tr}(I_{r})=r,$$

where: r—number of parameters of adopted model of vertical displacements, $I_{r}$—identity matrix of dimensions: *r* x *r*.

Whereas

(34) $$f_{2}={\left[E(\xi )\right]}^{T}\Theta E(\xi )=0^{T}\Theta 0=0.$$

Assuming that the square forms

(35) $$\xi^{T}\Theta \xi =\xi^{T}H^{T}C_{d}^{-1}H\xi ∼\chi_{f1=r}^{2},$$

and

(36) $${\hat{\delta }}^{T}C_{d}^{-1}\hat{\delta }∼\chi_{\nu =n-m_{r}}^{2},$$

are mutually independent, so their quotient has the *F*-Snedecora distribution,

(37) $$T_{G}=\frac{\frac{1}{r}\xi^{T}H^{T}C_{d}^{-1}H\xi }{\frac{1}{\nu }{\hat{\delta }}^{T}C_{d}^{-1}\hat{\delta }}=\frac{\xi^{T}H^{T}\sigma_{0}^{-2}P_{d}H\xi }{\frac{r}{\nu }{\hat{\delta }}^{T}\sigma_{0}^{-2}P_{d}\hat{\delta }}=\frac{\xi^{T}H^{T}P_{d}H\xi }{r\left(\frac{1}{n-m_{r}}{\hat{\delta }}^{T}P_{d}\hat{\delta }\right)}=\frac{\xi^{T}H^{T}P_{d}H\xi }{rm_{0}^{2}}.$$

Because Equation (37) adopts only positive values (the quotient of square forms), the probability that the variable $T_{G}$ will have the values less or equal to certain limited values, can be defined $F_{\alpha }\left(r,\nu \right)$. Hence

(38) $$P\left(T_{G}\leq F_{\alpha }\left(r,\nu \right)\right)=\alpha ,$$

or taking into consideration the Equation (37)

(39) $$P\left(\frac{\xi^{T}H^{T}P_{d}H\xi }{rm_{0}^{2}}\leq F_{\alpha }\left(r,\nu \right)\right)=\alpha .$$

Conducting the calculations in next measurement epochs, first the determination has to be done (as the difference of values of parameters gained from the calculations in epoch j and the theoretical values). Next step is the calculation of value of the square form $\xi^{T}H^{T}P_{d}H\xi$ and coefficient $m_{0}^{2}$. The results enable to provide global test $P\left(T_{G}\leq F_{\alpha }\left(r,\nu \right)\right)=\alpha$. The global test makes it possible to verify the correctness of fitting the examined space in the displaced model of object treated as the rigid body. It is worth to pay attention to the fact, that there can be a situation, where the global test will be fulfilled but individual parameters of vertical displacements’ model of examined object will exceed the values acceptable for them. In order to discover this transgressions, the statistical tests individual for each parameters (r=1), have to be conducted, with the use of the following dependence

(40) $$P\left(\frac{\xi_{i}^{T}H^{T}P_{d}H\xi_{i}}{m_{0}^{2}}\leq F_{\alpha }(r=1,\nu )\right)=\alpha ,$$

where: *i* means the values of individual coefficients of model gained from calculations $\xi_{i},\;i=a_{0},\;a_{1},\;a_{2}\ldots$. Assuming in the same way as in this paper $\xi_{i}\;(i=T_{Z},\;\varepsilon_{Y},\;\varepsilon_{X})$ the result is $\xi_{T_{Z}}={\left[T_{Z},\;0,\;0\right]}^{T}$, $\xi_{\varepsilon_{Y}}={\left[0,\;\varepsilon_{Y},\;0\right]}^{T}$, $\xi_{\varepsilon_{X}}={\left[0,\;0,\;\varepsilon_{X}\right]}^{T}$. 

## 3. Results

The practical analyses of theoretical consideration presented above were realized in two simulated variants.

Variant no. I—it was assumed that the HLS’s sensors are connected serially. 

Variant no. II—the separate connection for each HLS’s sensor with RS was assumed.

The results of observations *h* obtained from six sensors at starting epoch I (*j* = 0) and epoch II (*j*) are presented in Table 1.

In this work, in order to present the concept of accuracy assessment of the results coming from the HLS, the simplified calculation version, presented in Equation (16), was adopted. In further papers, the author is going to analyse the solutions gained from Equation (5) in detail. While conducting the calculations in the simplified version, it was assumed that the values of mean errors of readings from all sensors (RS and CPs) are the same and are equal to $m_{h}=0.01\;\mathrm{mm}$. Hence, the values of mean errors obtained from the Equation (16) are $m_{dZk}=\sqrt{m_{hi}^{2}+m_{hi+1}^{2}}=\sqrt{0.01^{2}+0.01^{2}}=0.014\;\mathrm{mm}$. The values gained $m_{dZk}$ enable to define the cofactors’ matrix $Q_{y}$, in the following form

$$\begin{array}{l} Q_{y}=diag\left(m_{dZ1}^{2},\;m_{dZ2}^{2},\;m_{dZ3}^{2},\;m_{dZ4}^{2},\;m_{dZ5}^{2},m_{dZ6}^{2}\right)=\\ =diag\left(0.01\sqrt{2},\;0.01\sqrt{2},\;0.01\sqrt{2},\;0.01\sqrt{2},\;0.01\sqrt{2},\;0.01\sqrt{2}\right) \end{array}$$

### 3.1. The Analysis of the Results Obtained from Variant No. I 

On the basis of the results presented in Table 1, the values $dZ_{k}=h_{i}-h_{i+1}$ were determined and are presented in Table 2, gained from six sensors at epoch I (*j*) and in epoch II (*j* = 0).

While creating the Equation (7), it was assumed that

$$A=\left[\begin{array}{llllll} 1 & 0 & 0 & 0 & 0 & 0\\ -1 & 1 & 0 & 0 & 0 & 0\\ 0 & -1 & 1 & 0 & 0 & 0\\ 0 & 0 & -1 & 1 & 0 & 0\\ 0 & 0 & 0 & -1 & 1 & 0\\ 0 & 0 & 0 & 0 & -1 & 1 \end{array}\right],-X=\left[\begin{array}{l} Z_{1}\\ Z_{2}\\ Z_{3}\\ Z_{4}\\ Z_{5}\\ Z_{6} \end{array}\right],-dz^{j=0}={\left[\begin{array}{l} -10.5\\ -6.0\\ 2.0\\ 7.3\\ 9.8\\ 1.0 \end{array}\right]}_{\;\mathrm{mm}},-dz^{j}={\left[\begin{array}{l} -10.9\\ -6.0\\ -5.1\\ 3.4\\ \;18.5\\ -2.8 \end{array}\right]}_{\;\mathrm{mm}}.$$

Hence, on the basis on Equation (8), the following results were gained:

$$\begin{array}{ll} & X^{j=0}={\left[-10.5,-16.5,-14.5,-7.2,\;2.6,\;3.6\right]}_{\mathrm{mm}}^{T},\\ \mathrm{and} & X^{j}={\left[-10.9,-16.9,-22.0,-18.6,\;-0.1,\;-2.9\right]}_{\mathrm{mm}}^{T} \end{array}$$

Whereas, the cofactors’ matrix of X parameters, obtained from Equation (11) has the following form

$$Q_{x}={\left[\begin{array}{llllll} 0.0001 & 0.0001 & 0.0001 & 0.0001 & 0.0001 & 0.0001\\ 0.0001 & 0.0002 & 0.0002 & 0.0002 & 0.0002 & 0.0002\\ 0.0001 & 0.0002 & 0.0003 & 0.0003 & 0.0003 & 0.0003\\ 0.0001 & 0.0002 & 0.0003 & 0.0004 & 0.0004 & 0.0004\\ 0.0001 & 0.0002 & 0.0003 & 0.0004 & 0.0005 & 0.0005\\ 0.0001 & 0.0002 & 0.0003 & 0.0004 & 0.0005 & 0.0006 \end{array}\right]}_{{\mathrm{mm}}^{2}}.$$

Determining the values of mean errors $m_{Zi}=\sqrt{Q_{X\left(i,i\right)}}$, (i,i=1,…,6—diagonal element of matrix) the following results were gained:

$$\begin{array}{l} m_{Z1}=0.010\;\mathrm{mm},\;m_{Z2}=0.014\;\mathrm{mm},m_{Z3}=0.017\;\mathrm{mm},m_{Z4}=0.020\;\mathrm{mm},m_{Z5}=0.022\;\mathrm{mm},\;\\ m_{Z6}=0.024\;\mathrm{mm}. \end{array}$$

The vector of vertical displacements of CPs: 1, 2, 3, 4, 5, 6: is $d=X^{j}-X^{j=0}={\left[d_{1}=-0.4,\;d_{2}=-0.4,\;d_{3}=-7.5,\;d_{4}=-11.4,\;d_{5}=-2.7,\;d_{6}=-6.5\right]}_{\;\mathrm{mm}}^{T}$. Whereas, the cofactors’ matrix $Q_{d}=Q_{X}{}_{j=0}+Q_{X}{}_{j}$ (for $Q_{X}{}_{j=0}=Q_{X}{}_{j}$) has the form of:

$$Q_{d}={\left[\begin{array}{llllll} 0.0002 & 0.0002 & 0.0002 & 0.0002 & 0.0002 & 0.0002\\ 0.0002 & 0.0004 & 0.0004 & 0.0004 & 0.0004 & 0.0004\\ 0.0002 & 0.0004 & 0.0006 & 0.0006 & 0.0006 & 0.0006\\ 0.0002 & 0.0004 & 0.0006 & 0.0008 & 0.0008 & 0.0008\\ 0.0002 & 0.0004 & 0.0006 & 0.0008 & 0.0010 & 0.0010\\ 0.0002 & 0.0004 & 0.0006 & 0.0008 & 0.0010 & 0.0012 \end{array}\right]}_{{\mathrm{mm}}^{2}}$$

Determining the values of mean errors $m_{di}=\sqrt{Q_{d\left(i,i\right)}}$, (i,i=1,…,6—diagonal element of matrix*)* obtained from the vertical displacements, the following results were gained:

$$\begin{array}{l} m_{d1}=0.014\;\mathrm{mm},\;m_{d2}=0.02\;\mathrm{mm},\;m_{d3}=0.024\;\mathrm{mm},\;m_{d4}=0.028\;\mathrm{mm},\;m_{d5}=0.032\;\mathrm{mm},\;\\ m_{d6}=0.035\;\mathrm{mm} \end{array}$$

### 3.2. The Analysus of the Results Obtained from Variant No. II

On the basis of the results presented in Table 1, the values $dZ_{k}=h_{RS}-h_{i}$ are determined and presented, Table 3.

In order to create the Equation (7), in variant no. II, it was assumed that

$$A=\left[\begin{array}{llllll} 1 & 0 & 0 & 0 & 0 & 0\\ 0 & 1 & 0 & 0 & 0 & 0\\ 0 & 0 & 1 & 0 & 0 & 0\\ 0 & 0 & 0 & 1 & 0 & 0\\ 0 & 0 & 0 & 0 & 1 & 0\\ 0 & 0 & 0 & 0 & 0 & 1 \end{array}\right],\;X=\left[\begin{array}{l} Z_{1}\\ Z_{2}\\ Z_{3}\\ Z_{4}\\ Z_{5}\\ Z_{6} \end{array}\right],\;dz^{j=0}={\left[\begin{array}{l} -10.5\\ -16.5\\ -14.5\\ -7.2\\ 2.6\\ 3.6 \end{array}\right]}_{\;\mathrm{mm}},\;dz^{j}={\left[\begin{array}{l} -10.9\\ -16.9\\ -22.0\\ -18.6\\ -0.1\\ -2.9 \end{array}\right]}_{\;\mathrm{mm}}.$$

While conducting calculations of Equation (8), the values of variables X were the same (which is not surprising) as in the variant no. I, namely:

$$\begin{array}{ll} & X^{j=0}={\left[-10.5,-16.5,-14.5,-7.2,\;2.6,\;3.6\right]}_{\mathrm{mm}}^{T},\\ \mathrm{and} & X^{j}={\left[-10.9,-16.9,-22.0,-18.6,\;-0.1,\;-2.9\right]}_{\mathrm{mm}}^{T} \end{array}$$

The cofactors’ matrix $Q_{x}$ of the X parameters determined from Equation (11) has the following form

$$Q_{x}={\left[\begin{array}{llllll} 0.0001 & 0 & 0 & 0 & 0 & 0\\ 0 & 0.0001 & 0 & 0 & 0 & 0\\ 0 & 0 & 0.0001 & 0 & 0 & 0\\ 0 & 0 & 0 & 0.0001 & 0 & 0\\ 0 & 0 & 0 & 0 & 0.0001 & 0\\ 0 & 0 & 0 & 0 & 0 & 0.0001 \end{array}\right]}_{{\mathrm{mm}}^{2}}.$$

Hence, the values of men errors $m_{Zi}=\sqrt{Q_{X\left(i,i\right)}}$ are the same and equal to respectively:

$$\begin{array}{l} m_{Z1}=0.010\;\mathrm{mm},\;m_{Z2}=0.010\;\mathrm{mm},m_{Z3}=0.010\;\mathrm{mm},m_{Z4}=0.010\;\mathrm{mm},m_{Z5}=0.010\;\mathrm{mm},\;\\ m_{Z6}=0.010\;\mathrm{mm}\; \end{array}$$

The vector of vertical displacements $d=X^{j}-X^{j=0}$ has the same components as in variant no. I, $d={\left[d_{1}=-0.4,\;d_{2}=-0.4,\;d_{3}=-7.5,\;d_{4}=-11.4,\;d_{5}=-2.7,\;d_{6}=-6.5\right]}_{\;\mathrm{mm}}^{T}$. The cofactors’ matrix $Q_{d}=Q_{X}{}_{j=0}+Q_{X}{}_{j}$ (for $Q_{X}{}_{j=0}=Q_{X}{}_{j}$) has the following form

$$Q_{d}={\left[\begin{array}{llllll} 0.0002 & 0 & 0 & 0 & 0 & 0\\ 0 & 0.0002 & 0 & 0 & 0 & 0\\ 0 & 0 & 0.0002 & 0 & 0 & 0\\ 0 & 0 & 0 & 0.0002 & 0 & 0\\ 0 & 0 & 0 & 0 & 0.0002 & 0\\ 0 & 0 & 0 & 0 & 0 & 0.0002 \end{array}\right]}_{{\mathrm{mm}}^{2}}$$

Determining the values of mean errors $\left(m_{di}=\sqrt{Q_{d\left(i,i\right)}}\right)$ obtained from the vertical displacements, the following results were gained:

$$\begin{array}{l} m_{d1}=0.014\;\mathrm{mm},\;m_{d2}=0.014\;\mathrm{mm},\;m_{d3}=0.014\;\mathrm{mm},\;m_{d4}=0.014\;\mathrm{mm},\;m_{d5}=0.014\;\mathrm{mm},\;\\ m_{d6}=0.014\;\mathrm{mm} \end{array}$$

The modelling of vertical displacements of examined object was made for both variants. The rectangular values presented in Table 4 were used in calculations. 

Using Equation (21) the following equations were formulated

$$\begin{array}{l} \delta_{1}-0.4=T_{Z}+0\varepsilon_{Y}-40\varepsilon_{X}\\ \delta_{2}-0.4=T_{Z}+0\varepsilon_{Y}-80\varepsilon_{X}\\ \delta_{3}-7.5=T_{Z}+0\varepsilon_{Y}-120\varepsilon_{X}\\ \delta_{4}-11.4=T_{Z}+30\varepsilon_{Y}-120\varepsilon_{X}\\ \delta_{5}-2.74=T_{Z}+30\varepsilon_{Y}-80\varepsilon_{X}\\ \delta_{6}-6.5=T_{Z}+30\varepsilon_{Y}-40\varepsilon_{X} \end{array}\}\Rightarrow H=\left[\begin{array}{lll} 1 & 0 & -40\\ 1 & 0 & -80\\ 1 & 0 & -120\\ 1 & 30 & -120\\ 1 & 30 & -80\\ 1 & 30 & -40 \end{array}\right],\;t=\left[\begin{array}{l} T_{Z}\\ \varepsilon_{Y}\\ \varepsilon_{X} \end{array}\right],\;\delta =\left[\begin{array}{l} 0.4\\ 0.4\\ 7.5\\ 11.4\\ 2.7\\ 6.5 \end{array}\right].$$

Equations (24)–(26) were used for calculations. The calculations’ results are presented in Table 5 and Table 6.

The comparison of the corrections’ values ${\hat{\delta }}_{i}$ to vertical displacements $d_{i}$ obtained for both variants are presented in Table 6. The corrections’ values enable to calculate the coefficients $m_{0}^{2}$ for both variants. These coefficients are, respectively, for variant no. I $m_{0}^{2}=0.0596$ and for variant no. II $m_{0}^{2}=0.0594$. For accuracy assessment of adopted model of vertical displacements, the global test defined in Equation (37) was used. For more detailed analyses it was assumed that α=0.05 and the values of degrees of freedom r = 6, ν = 3. For this data values, the critical value $F_{\alpha }$ = 8.94 was determined from the distribution tables. The Table 7 presents the results of global test. The local tests of coefficients’ of displacements’ model for both analyzed variants were conducted, using Equation (40). For local tests the following assumptions were made according to the theoretical assumptions r=1, ν=6. Hence, the critical value of *F*-Snedecore distribution $F_{\alpha }=5.99$, (α=0.05). The obtained calculations’ values are presented in Table 7.

From the data presented in Table 7, it can be noticed that the global test was positive in both analyzed variants. The values obtained $T_{G}=2.86$ (variant no. 1) and $T_{G}=2.81$ (variant no. 2) and they were less that the critical value $F_{\alpha }=8.94$. However, after conducting the tests for individual values of parameters of vertical displacements’ model, the parameter $\varepsilon_{X}$, for which tested statistics were $T_{\varepsilon X}=21.18$ (variant no. 1) and $T_{\varepsilon X}=21.21$ (variant no. 2), exceeded the critical value of test $T_{\alpha }=5.99$. The obtained value provides the information about inaccurate fitting of the vertical displacements’ model in the examined object treated as the rigid body. Hence, the conclusion is that there is a need to look for other model describing the vertical displacements. Searching the most beneficial model is the individual problem defined for each examined object. It is also more technical than the scientific problem, hence, in this paper, it will not be further analysed. 

## 4. Discussion

On the basis of the example presented in this paper regarding to the simplified version of model denoted as Equation (15) and its accuracy assessment in the form of Equation (16), it is impossible to define too general conclusions. However, it is worth to mention that these kind of constructions can be found in practical applications. Summing up the gained results, it must be stated that in both considered variants, the same values of vertical displacements of CPs $d_{i}$, (*i* = 1,…,6) were obtained. However, the accuracy analyses represented by the covariance matrices are different. While analysing the obtained results, it is noticeable that in variant no. II the assessment of mean errors of vertical displacements were more precise than in variant no. I. The attention should also be paid to the formula of cofactors’ matrix $Q_{d}$ (variant no. I) of vertical displacements, where there are the covariance assessments between the displacements $d_{i}$. It testifies that the variables are correlated. Calculating the linear correlation coefficient $\rho =\frac{\mathrm{cov}(d_{i},d_{j})}{\sigma_{di}\sigma_{dj}}$, using the cofactors’ matrix $Q_{d}$ (variant no. I), the results are presented in Table 8.

While analysing data presented in Table 8, it can be noticed that the linear correlation coefficient ρ grows with the decreasing number of CPs. The less CPs, the bigger the linear correlation between points is. 

Whereas, the formula of cofactors’ matrix (variant no. II) shows that the displacements $d_{i}$ are not correlated to each other. On the basis of the presented calculations and the defined remarks, it can be stated that more advantages for a solution (taking into consideration the determinations’ accuracy) is variant no. II. This is the one where the individual sensors of CPs are connected directly with the RS. Because in this situation there is a need for frequent repeats of long and independent connections between sensors, the consequence of this solution can be higher costs of HLS.

Another important usage of obtained values of cofactors’ matrix $Q_{d}$ is also the possibility to apply them in geostatistics, mainly in the subject of vertical displacements’ modelling using the Kriging method. On the basis of empirical semi-variogram (determined from the linear correlation function), the theoretical model of semi-variogram can be fitted to it. This model, together with determined coordinates X,Y, can support the process of making decisions regarding these areas of examined body, where HLS sensors were not located. 

As it was mentioned before, this paper does not bring up the topic represented in Equation (3) and accuracy analysis defined in Equation (5). More detailed researches regarding these issues will be presented in further papers of the author. Thus, the author invites everyone to a scientific cooperation who are interested in the problems presented in this paper. 

## Figures and Tables

**Table 1:** The results h obtained from epoch I and epoch II.

Sensors’ Number	Epoch I (*j*) $\mathrm{Observations}\;h_{i}\;[\mathrm{mm}]$	Epoch II (*j* = 0) $\mathrm{Observations}\;h_{i}\;[\mathrm{mm}]$
RS	52.3	44.2
1	63.2	54.7
2	69.2	60.7
3	74.3	58.7
4	70.9	51.4
5	52.4	41.6
6	55.2	40.6

**Table 2:** The results $dZ_{k}$ obtained from epoch I and epoch II.

Number of Height Difference	Epoch I (*j*) $dZ_{k}[\mathrm{mm}]$	Epoch II (*j = 0*) $dZ_{k}\;[\mathrm{mm}]$
$dZ_{1}$(RS-1)	−10.9	−10.5
$dZ_{2}$(1-2)	−6.0	−6.0
$dZ_{3}$(2-3)	−5.1	2.0
$dZ_{4}$(3-4)	3.4	7.3
$dZ_{5}$(4-5)	18.5	9.8
$dZ_{6}$(5-6)	−2.8	1.0

**Table 3:** The results $dZ_{k}$ obtained from epoch I and epoch II.

Number of Heights Difference	Epoch I (*j*) $dZ_{k}\;[\mathrm{mm}]$	Epoch II (*j = 0*) $dZ_{k}\;[\mathrm{mm}]$
$dZ_{1}$(RS-1)	−10.9	−10.5
$dZ_{2}$(RS-2)	−16.9	−16.5
$dZ_{3}$(RS-3)	−22.0	−14.5
$dZ_{4}$(RS-4)	−18.6	−7.2
$dZ_{5}$(RS-5)	−0.1	2.6
$dZ_{6}$(RS-6)	−2.9	3.6

**Table 4:** The rectangular values of sensors X, Y [m].

**Sensors’ Number**	**X [m]**	**Y [m]**
RS	0.0	0.0
1	0.0	40.0
2	0.0	80.0
3	0.0	120.0
4	30.0	120.0
5	30.0	80.0
6	30.0	40.0

**Table 5:** The obtained model’s parameters and their mean errors.

Parameters	Variant No. I	Variant No. II
${\hat{\varepsilon }}_{Y}$	$-92^{cc}$ $m_{\hat{\varepsilon }Y}=7.7^{cc}$	$-87^{cc}$ $m_{\hat{\varepsilon }Y}=7.7^{cc}$
${\hat{\varepsilon }}_{X}$	$48^{cc}$ $m_{\hat{\varepsilon }X}=3.6^{cc}$	$48^{cc}$ $m_{\hat{\varepsilon }X}=3.6^{cc}$
${\hat{T}}_{Z}$	0.08 mm $m_{\hat{T}z}=0.5\;mm$	3.2 mm $m_{\hat{T}z}=0.5\;\mathrm{mm}$

**Table 6:** The obtained corrections $\hat{\delta }$ [mm].

Corrections	Variant No. I	Variant No. II
${\hat{\delta }}_{1}$	0.0008	0.0006
${\hat{\delta }}_{2}$	−0.0022	−0.0024
${\hat{\delta }}_{3}$	0.0019	0.0017
${\hat{\delta }}_{4}$	0.0014	0.0015
${\hat{\delta }}_{5}$	−0.0043	−0.0042
${\hat{\delta }}_{6}$	0.0025	0.0026

**Table 7:** Tests.

Tests	Variant No. I	Variant No. II
global:	$T_{G}=2.86$	$T_{G}=2.81$
local: $\varepsilon_{Y}$	$T_{\varepsilon Y}=4.78$	$T_{\varepsilon Y}=4.25$
local: $\varepsilon_{X}$	$T_{\varepsilon X}=21.18$	$T_{\varepsilon X}=21.21$
local: $T_{Z}$	$T_{Z}=5.71$	$T_{Z}=5.28$

**Table 8:** The values of the linear correlation coefficient ρ.

CPs Number	1	2	3	4	5	6
1	x	0.71	0.60	0.51	0.45	0.41
2		x	0.83	0.71	0.62	0.57
3			x	0.89	0.78	0.71
4				x	0.89	0.81
5	S y	m m	e t	r y	x	0.89
6						x

## Data Availability

No new data were created or analyzed in this study. Data sharing is not applicable to this article.
